# Deciphering Egyptian Hieroglyphs: Towards a New Strategy for Navigation in Museums

**DOI:** 10.3390/s17030589

**Published:** 2017-03-14

**Authors:** Jaime Duque-Domingo, Pedro Javier Herrera, Enrique Valero, Carlos Cerrada

**Affiliations:** 1 Depto.de Ingeniería de Software y Sistemas Informáticos, ETSI Informática, UNED. C/Juan del Rosal, 16, 28040 Madrid, Spain; pjherrera@issi.uned.es; 2 School of Energy, Geoscience, Infrastructure and Society, Heriott-Watt University, Edinburgh EH14 4AS, UK; e.valero@hw.ac.uk

**Keywords:** Egyptian hieroglyphs, edge detection, region identification, object recognition

## Abstract

This work presents a novel strategy to decipher fragments of Egyptian cartouches identifying the hieroglyphs of which they are composed. A cartouche is a drawing, usually inside an oval, that encloses a group of hieroglyphs representing the name of a monarch. Aiming to identify these drawings, the proposed method is based on several techniques frequently used in computer vision and consists of three main stages: first, a picture of the cartouche is taken as input and its contour is localized. In the second stage, each hieroglyph is individually extracted and identified. Finally, the cartouche is interpreted: the sequence of the hieroglyphs is established according to a previously generated benchmark. This sequence corresponds to the name of the king. Although this method was initially conceived to deal with both high and low relief writing in stone, it can be also applied to painted hieroglyphs. This approach is not affected by variable lighting conditions, or the intensity and the completeness of the objects. This proposal has been tested on images obtained from the Abydos King List and other Egyptian monuments and archaeological excavations. The promising results give new possibilities to recognize hieroglyphs, opening a new way to decipher longer texts and inscriptions, being particularly useful in museums and Egyptian environments. Additionally, devices used for acquiring visual information from cartouches (i.e., smartphones), can be part of a navigation system for museums where users are located in indoor environments by means of the combination of WiFi Positioning Systems (WPS) and depth cameras, as unveiled at the end of the document.

## 1. Introduction

Egyptian writing is complex and is based on more than 800 characters, named hieroglyphs. They were initially considered pictograms representing words, but Champollion deciphered the writing system and discovered that in fact, they are characters. Although most of hieroglyphs are sounds composed of one, two or three phonemes, some of them are pictograms [1]. The name of the monarchs, named pharaohs, was composed of a box with usually rounded corners, called cartouche, and a word inside. This word usually can be translated as a phrase, e.g., Tutankhamen would be “Living image of god Amen”.

During the last decades, the advancements in the field of computer vision have facilitated the study of ancient writing systems, especially regarding the automatic recognition of characters. For instance, several methods have been developed to detect edges and recognize objects [2,3,4]. With respect to the Egyptian writing system, several authors have studied the hieroglyph detection problem. The difference between the intensity values of the hieroglyphs and the rest of the surface is used in [5] to extract hieroglyphs. Shape descriptors are used for hieroglyph retrieval purposes in [6,7]. The shape context descriptor proposed by [8] takes as input a finite set of *n* points, which represent the contours of a given shape, and describes it as a set of *n* histograms. With these histograms, the difference between a pixel in one contour and the rest of the pixels in the other contour is evaluated. Locating the minor difference between the pixels of the first contour and the second, the global difference between the two contours is evaluated. This descriptor is used to obtain Mayan hieroglyphs in [6]. The main limitation of this method is to consider that hieroglyphs are complete. It would be more effective if edges could be entirely extracted. However, in some cases, ancient texts are not well preserved and these defects are reflected as noise in the images. An evaluation of the performance of three shape-based contextual descriptors (Shape Context—SC, Generalized Shape Context—GSC, and Histogram of Orientation Shape-Context—HOOSC) using two datasets of syllabic Maya hieroglyphs is presented in [7]. SC and GSC proved to generate reliable descriptions of Maya hieroglyphs whereas HOOSC proved to be more robust. According to these studies, hieroglyphs may be extracted and a shape descriptor applied to obtain each character. Other recent works are focused on the identification of handwritten hieroglyphic transcriptions drawn in plain (i.e., black or white) backgrounds and are based on text mining and natural language processing techniques [9,10]. Nevertheless, hieroglyphic texts encoding has been reported to be time-consuming.

**Figure 1:**
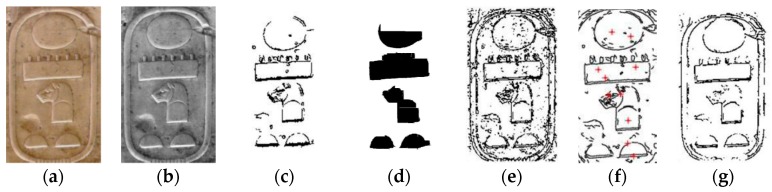
(**a**) Original image; (**b**) Monochrome image; (**c**) Sobel edge detection; (**d**) regions by frontier; (**e**) SUSAN algorithm; (**f**) Circle Hough Transform to obtain most-probable circles; (**g**) curvature salience after 20 iterations.

In order to solve the above-mentioned problems, other strategies have been proposed. It was initially considered extracting the hieroglyphs using a conversion to grey scale and applying a threshold and the Hu moments [11,12,13]. It could be a right method if the color of the hieroglyphs was different. However, the hieroglyphs have the same color than other parts of the cartouche and the extraction process becomes difficult. Secondly, it was tried to use a strategy of regions by frontier based on the gradient, the Laplacian and the Hu moments [2]. To use this proposal the edges were detected using Sobel, Canny and Susan methods [2,14,15]. The problem with this another method is that sometimes there is noise or edges that are not well defined. Figure 1 shows examples of application of the methods evaluated on the images used in this work. The Generic Hough Transform (GHT) is a well-known method that can be used to obtain a concrete object using its edge [16]. However, the main drawback is that it only considers the points that exactly match with the model. If there is a big difference between the points of the edge and the edge of the image, it returns non-valid results as one can see in Figure 1f. The hieroglyph represented in Figure 1a was not properly detected because there are no perfect circles in the image. The curvature and structural salience method tries to extract the regions with special interest [17]. This method was tested on edges of cartouches for extracting the hieroglyphs, but the results were similar to apply a threshold to the edges as can be seen in Figure 1g.

**Figure 2:**
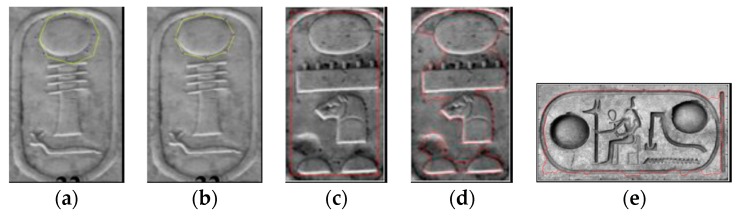
(**a**,**b**) AAM method applied to hieroglyph images; Active Contour Algorithm applied to obtain the edge of the Egyptian hieroglyphs (**c**) initially; (**d**) after 1000 iterations; (**e**) Active Contour Algorithm applied to obtain the edge of the Egyptian cartouche after 300 iterations.

Other methods as Statistical Shape Models (SSM), Active Shape Models (ASM), and Active Appearance Models (AAM) are used to find objects with a similar shape using key points or landmarks and the textures between these landmarks [18,19]. These methods work better when there is a clear difference between the object and the rest of the image. In this work, the hieroglyphs have the same color than the rest of the cartouche, and the edges are very soft. Figure 2a,b shows an example of AAM method. Other approach implements a skeleton search using a Skeletal Shape Model for object segmentation and recognition that works directly on real images [20]. A fragment-based generative model for shape that is capable of generating a wide variation of shapes as instances of a given object category is proposed. It develops a progressive selection mechanism to search among the generated shapes for the category instances that are present in the image. This method seems to be robust against spurious edges, missing edges, and accidental alignment between the image edges and the shape boundary contour. The segmentation of the object using global holistic properties of object shape is proposed in [21]. Concretely, it proposes a figure/ground segmentation method for extraction of image regions that resemble the global properties of a model boundary structure and are perceptually salient. Its shape representation, called the chordiogram, is based on geometric relationships of object boundary edges, but this work takes into account the perceptual saliency in favor of coherent regions distinct from the background. A new weighting function, which helps constructing words representations for detection of Maya hieroglyphs is introduced in [22]. This weighting function depends on the ratio of intersection of the local descriptors, and their respective distances to the center of the bounding box that is under evaluation. The Active Contours Algorithm was also tested in order to detect the edges of the Egyptian cartouches used in this work [23,24]. Figure 2c–e shows an example. Its performance was not suitable in terms of time.

Aiming to deal with the above-mentioned limitations, this work presents a new method to recognize Egyptian cartouches based on several computer vision techniques. This paper is organized as follows: Section 2 describes the approach developed for hieroglyphs interpretation: firstly, the localization of the cartouche in the initial picture is carried out; next, the hieroglyphs are extracted and identified; and lastly, the symbols are interpreted. Section 3 analyses the performance of the proposed method. Finally, Section 4 presents the conclusions and future work.

## 2. Overview of the Proposed Method to Interpret Hieroglyphs

**Figure 3:**
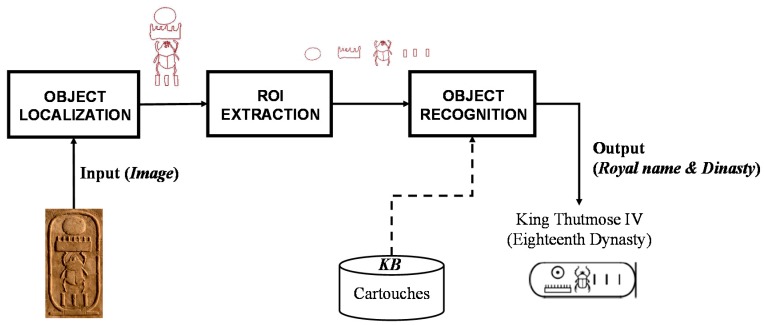
The proposed approach consists of three stages.

This section illustrates the strategy developed to localize and recognize the objects of interest in the image (i.e., hieroglyphs). As previously mentioned, this approach is based on different techniques used in computer vision and consists in the following three stages: (1) localization of the object (i.e., the cartouche) in the image and deletion of object’s contour; (2) extraction of regions of interest (ROIs), which are the hieroglyphs; (3) recognition of cartouches considering the identification of each hieroglyph. Figure 3 shows the described process. First of all, it is worth mentioning some factors that should be considered for an automatic visual analysis and interpretation of the Egyptian writing:

1. Texts were written to be read from the left to the right or from the right to the left.
2. Egyptian scribes were able to write in different materials: stone, wood, faience, papyrus, gold, bronze, etc. Hieroglyphs were even painted.
3. Differences between hieroglyphs in different texts or materials are not remarkable, as a similar model was used.
4. Texts were written in both low-relief and high-relief art. In low-relief, hieroglyphs were incised in the stone. In high-relief, the rest of the cartouche was incised.
5. Most texts preserved until the present day have suffered the effects of time, exposure and even vandalism.

**Figure 4:**
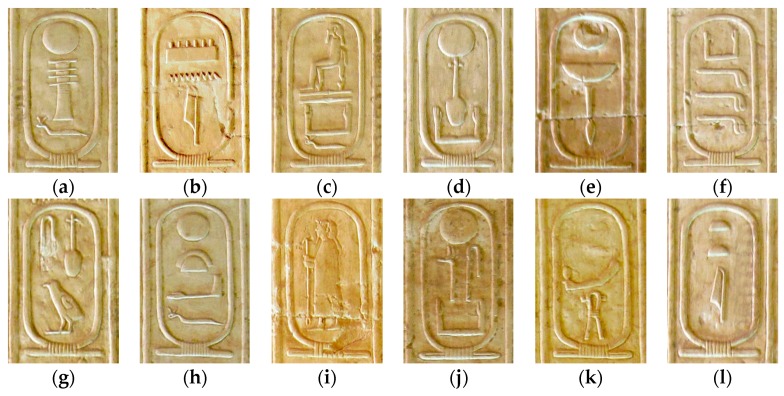
A set of cartouches from the Abydos King List: (**a**) Djedefre; (**b**) Menes; (**c**) Shepseskaf; (**d**) Neferkara I; (**e**) Mentuhotep II; (**f**) Raneb; (**g**) Sneferu; (**h**) Khafra; (**i**) Semerkhet; (**j**) Userkare; (**k**) Djoser; (**l**) Sekhemkhet.

Although the presented approach has been designed to be applied to cartouches written in stone, it can be also employed for painted hieroglyphs. Besides, it can be used for low-relief or high-relief writing. The state of preservation of the material (usually stone) as well as missing parts have an important effect in the final result. Thus, images can be noisy or intermediate results, like extracted contours, can correspond to incomplete hieroglyphs, making the recognition process more complicated. Figure 4 shows a set of images used in this work.

### 2.1. Localization of Cartouches in Images

**Figure 5:**
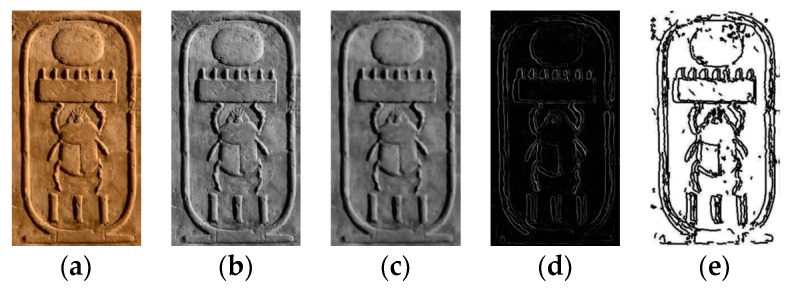
(**a**) Original image; (**b**) grayscale image; (**c**) median filter; (**d**) Canny edges; (**e**) threshold ISODATA inverted.

The localization process consists of seven steps where various image processing techniques are used. The main challenge of this stage is to localize the contour of each cartouche in the image. Once the cartouche is localized, the process deletes the contour. In the following, the mentioned localization steps are detailed:

*Step 1*. The input image, shown in Figure 5a, is transformed from RGB to grayscale (Figure 5b), using the luma, in-phase, quadrature (YIQ) color space as illustrated in Equation (1). Note that this system is usually employed in color image processing transformations [2].

(1) Y=0.299⋅R+0.587⋅G+0.114⋅B

*Step 2*. A median filter and a morphological erosion are applied. These filters are used to remove the noise without reducing the edges. Figure 5c presents the result of these operations.

*Step 3*. A Canny edge detector is applied to obtain the edges of the image [25] (see Figure 5d), being this one of the most powerful edge-detection methods.

*Step 4*. The Iterative Self-Organizing Data Analysis Technique (ISODATA) threshold is used to detect the most important edges, as illustrated in Figure 5e [26]. The objective is to split non-homogeneous regions into two sub-regions (objects and background) as follows:

*(a)*  A threshold *T* is set to a random value
*(b)*  The image is binarized using *T*
*(c)*  The mean values $\mu_{1}$, $\mu_{2}$ of the two sub-regions (objects and background) generated with *T* are obtained. $\mu_{1}$ is the mean value of all values under or equal to *T*. $\mu_{2}$ is the mean value of all values above *T*
*(d)*  A new threshold is calculated as $T=\left(\mu_{1}+\mu_{2}\right)/2$
*(e)*  Repeat steps *(b)* to *(d)* until *T* stops changing its value.

**Figure 6:**
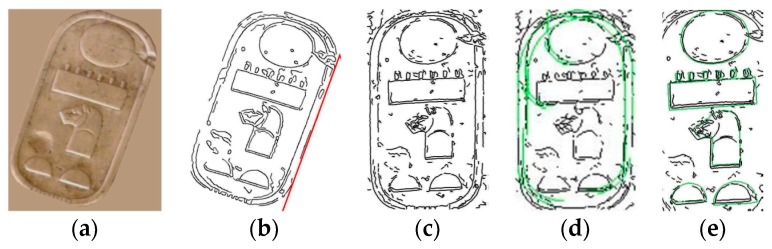
(**a**) Original image; (**b**) Longest border of the cartouche; (**c**) Cartouche after orientation correction; (**d**) Recognition of the cartouche borders; (**e**) Extraction of the regions of interest.

*Step 5.* If an image contains a rotated cartouche, as exemplified in Figure 6a, an alignment process is optionally performed to place their main directions parallel to the vertical and horizontal axes (Figure 6b). Following the procedure to detect lines in picture presented by [27], the longest line of the cartouche’s edge is obtained, as well as its orientation.

*Step 6.* In order to extract the hieroglyphs of the image, a search of the four corners of the cartouche is accomplished. A further explanation of this technique can be found in *Step 8*, as this is also applied to identify hieroglyphs. The image is reduced to a maximum width, keeping the aspect rate.

**Figure 7:**
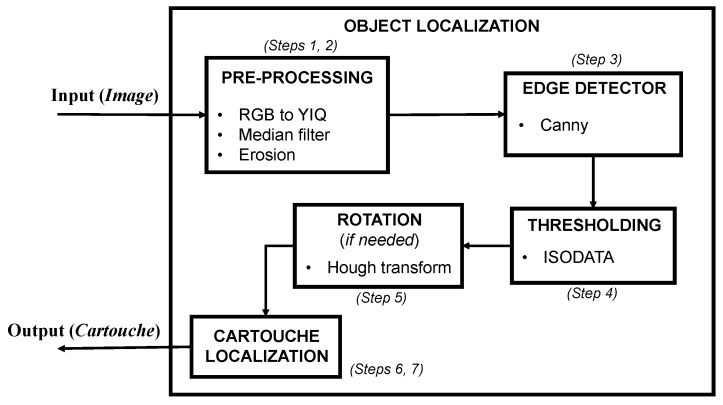
Scheme of the object localization process.

*Step 7.* Once the limits of the cartouche are calculated, each Region of Interest (ROI) will be extracted. In this work, each ROI corresponds to a hieroglyph and is always located within a cartouche in the image. Figure 6d,e shows the recognition of the cartouche borders and the ROI extraction process, respectively. Differences between hieroglyphs and cartouches are not remarkable because a similar model was used by Egyptian scribes. The ROI extraction process is described in Section 2.2. As a summary, Figure 7 shows a scheme of the object localization process described in this section.

### 2.2. Extraction and Identification of Hieroglyphs

At this point, the cartouche has been localized and the hieroglyphs will be individually extracted and identified. During the identification process, Chamfer [28] and Hausdorff [29] distances have been calculated between pairs of objects (one extracted by our algorithm from an image and the other coming from a database).

On one hand, Chamfer distance is calculated by means of the sum of the minimum distance for each point of the edge of an object *A* and a point of the another edge of object *B*. Chamfer distance considers all the points of the first object for obtaining the total sum:

(2) $$d_{C}\left(A,B\right)=\sum_{a\in A}\min_{b\in B}\left\|a-b\right\|$$

On the other hand, Hausdorff distance is different because it only considers the greatest of all the distances from each point in the contour of object *A* to the closest point in the contour of object *B*. It is obtained as in Equation (3); firstly, calculating the minimal distance for each point of one edge to another edge and finally, selecting the maximum of them:

(3) $$d_{H}\left(A,B\right)=\max_{a\in A}\left[\min_{b\in B}\left\|a-b\right\|\right]$$

**Figure 8:**
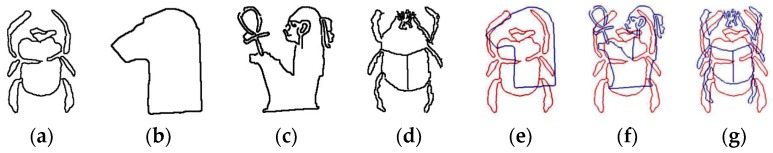
(**a**) Hieroglyph from a database to be used as a reference; (**b**–**d**) Hieroglyphs extracted from several cartouches; (**e**–**g**) Overlapped images.

Table 1 shows the results of Chamfer and Hausdorff distances once compared the hieroglyph in Figure 8a to the three hieroglyphs in Figure 8b–d. One can observe that lower distances, and therefore a better matching, are achieved for the hieroglyph in Figure 8d. Following the technique previously mentioned, an approximation to the Euclidean distance using Chamfer method is used. The extraction and identification of each hieroglyph is carried out by means of the following steps.

**Figure 9:**
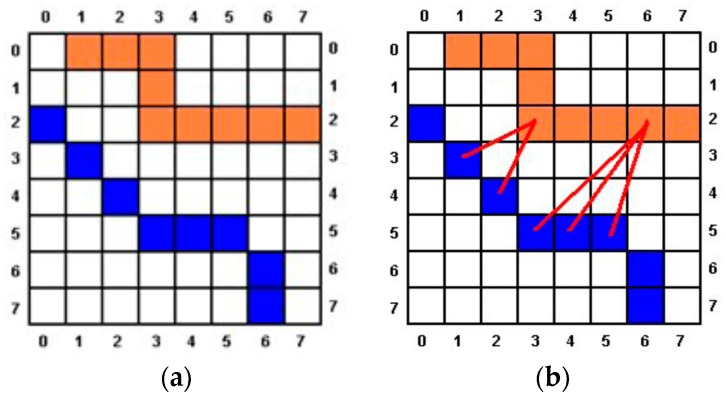
Distance between points *P_h_* (**a**) and points *P_c_* (**b**).

*Step 8.* First, the width of the hieroglyph is considered to be 8/10 of the width of the image, keeping the aspect rate. A hieroglyph is extracted when at least *T*% of the points of the hieroglyph’s contour satisfy that:

*(a)*  The Chamfer distance between each point of the hieroglyph’s contour (*p_h_*) and each point of the cartouche’s contour (*p_c_*) is lower than *d*. *d* is set to 3 pixels for a cartouche of 100 pixels width. Figure 9, if the hieroglyph’s contour is marked in orange and the cartouche’s contour one is colored in blue, the distance between *p_h_*(2,3) and the image is 2 because *p_c_*(3,1) and *p_c_*(4,2) are the closest points of the cartouche, and the distance to them is 2. The minimum distance from *p_h_*(2,6) to the cartouche is higher than 3 because the closest points are *p_c_*(5,3), *p_c_*(5,4) and *p_c_*(5,5). To calculate the distance it can be used a convolution mask over the central point, and it will be increased each iteration
*(b)*  The angle of the contour line in *p_c_* minus the angle of the contour line in *p_h_* is less than *Max_Angle*. The contours have been obtained by using the Canny algorithm, which produces non-maximum suppression (the width of the contour is 1 pixel), so the contours that include *p_c_* and *p_h_* have 1 pixel width. *Max_Angle* is calculated as in (4):
  (4) Max_Angle=(100−T)⋅π2100

The angle ϕ of a point respect to their neighboring points is obtained as in (5) where *A* is the number of pixels in the region, *x_i_* are the *x* positions with pixels, and *y_i_* are the *y* positions with pixels. The gradient of a point is not used in this proposal because the gradient gives the angle change of a point in a segment:

(5) $$\begin{array}{l} S_{x}=\sum x_{i}S_{y}\;;\;S_{y}=\sum y_{i}\\ S_{xx}=\sum {x_{i}}^{2}S_{yy}\;;\;S_{yy}=\sum {y_{i}}^{2}\;;\;S_{xy}=\sum x_{i}y_{i}\\ M_{xx}=S_{xx}-\frac{{S_{x}}^{2}}{A}\;;\;M_{yy}=S_{yy}-\frac{{S_{y}}^{2}}{A}\;;\;M_{xy}=S_{xy}-\frac{S_{x}\cdot S_{y}}{A}\\ \phi =\tan^{-1}\left[\frac{M_{xx}-M_{yy}+\sqrt{{\left(M_{xx}-M_{yy}\right)}^{2}+4\cdot {M_{xy}}^{2}}}{2\cdot M_{xy}}\right] \end{array}$$

If a point *p_c_* satisfied *(a)* and *(b)*, *P_c_* is assigned to *p_h_*, and *p_h_* is assigned to *p_c_*. If a cartouche’s point (*p_ck_*) has been assigned to a hieroglyph’s point (*p_hk_*) then *p_ck_* is not used again in the process.

The processed region is identified as a hieroglyph if it is satisfied the *T* percentage of the points assigned to the image and the number of continue lines that compose the contour assigned to the image (i.e., *p_c_* points that have been linked to *p_h_*) is lower than *L* (*L* = 20 in this work). This restriction prevents from some detected cases where noise provoke errors in detection.

*Step 9.* The size of the region is reduced in *R* pixels (*R* = 2 pixels in this work) and the step 8 is repeated in order to find the same region in the image. See the two bread bun shape hieroglyphs at the bottom of Figure 6e. The region’s size is reduced until its size is less than a percentage *Q* of the cartouche’s width (concretely *Q* = 33).

*Step 10.* If there are candidates to be labeled as hieroglyphs, the object with more coincidences is the accepted, i.e., the object with highest number of assigned points.

**Figure 10:**
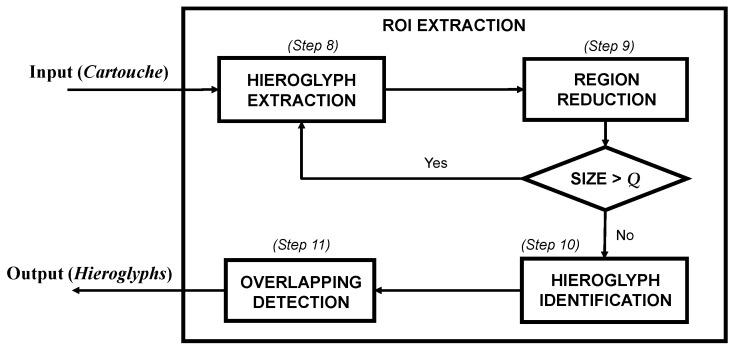
Scheme of the ROI extraction process.

*Step 11.* Once all the candidates have been identified, a new searching process is launched to detect possible small hieroglyphs contained by bigger ones. To do this, it is checked if there are two objects sharing an area of at least 33%. In this case, the smaller object is rejected. This overlapping is calculated by considering each object as a box that contains the object itself, being this limited by the detected edges. As a summary, Figure 10 shows a scheme of the hieroglyphs extraction process described in this section.

### 2.3. Interpretation of Cartouches

**Figure 11:**
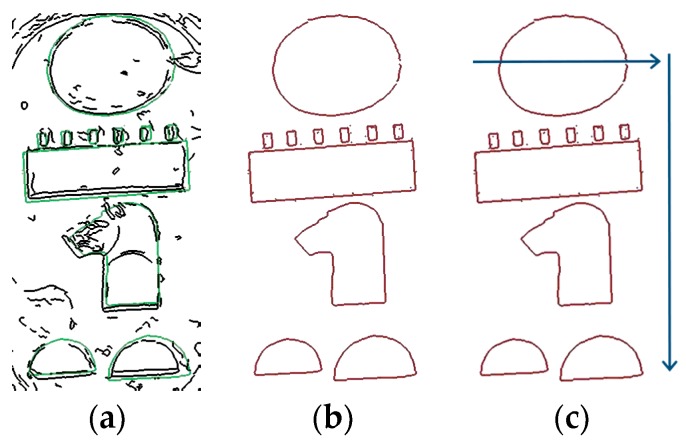
(**a**) Hieroglyphs extraction; (**b**) Hieroglyphs identification; (**c**) Reading sequence.

After analyzing the contents of the evaluated cartouche, a list of hieroglyphs with their positions is delivered. According to the procedures about Egyptian writing interpretation presented at the beginning of this section (i.e., studying the image from top to bottom and from left to right), the sequence of the hieroglyphs is read (see Figure 11).

Note that ancient Egyptian texts can be also written from right to left, so this option is also considered. The list of labeled hieroglyphs obtained in the previous steps are analyzed to figure out the name of the represented monarch. As these symbols can be read from left to right or vice versa, several combinations should be considered. Aiming to arrange the symbols in a proper manner and obtain a coherent result (i.e., name and dynasty), Levenshtein distance is calculated for each group of symbols. Levenshtein distance is a string metric used to measure the difference between two text sequences $s=s_{1}s_{2}s_{3}\ldots s_{n}$ and $t=t_{1}t_{2}t_{3}\ldots t_{m}$, e.g., the distance between two words is the minimum number of single-character edits (i.e., additions, subtractions or substitutions) required to change one word into the other [30]. In this work, each hieroglyph corresponds to a character in the string, e.g., the distance between RA-MN-PHTY-T-T (Ramesses I), and RA-MN-HPR (Thutmose III) is 3 (one substitution and two additions). The Levenshtein distance for each name is calculated as in Equation (6), and the name with minor value is returned:

(6) $$d_{s,t}\left(i,j\right)=\{\begin{array}{ll} \max\left(i,j\right) & \mathrm{if}\;\min\left(i,j\right)=0\\ \min\{\begin{array}{l} d_{s,t}\left(i-1,j\right)+1\\ d_{s,t}\left(i,j-1\right)+1\\ d_{s,t}\left(i-1,j-1\right)+I_{s,t}\left(i\neq j\right) \end{array} & \mathrm{otherwise} \end{array}$$

where $d_{s,t}\left(i,j\right)$ is the distance between the first hieroglyph *i* of the text sequence *s* and the first hieroglyph *j* of the string *t*; and I(i≠j) is the indicator function (equal to 0 when i=j and equal to 1 otherwise). The first expression in the bracket after *min* corresponds to subtraction, the second one to addition and the third one to matching (if the respective hieroglyphs are similar).

**Figure 12:**
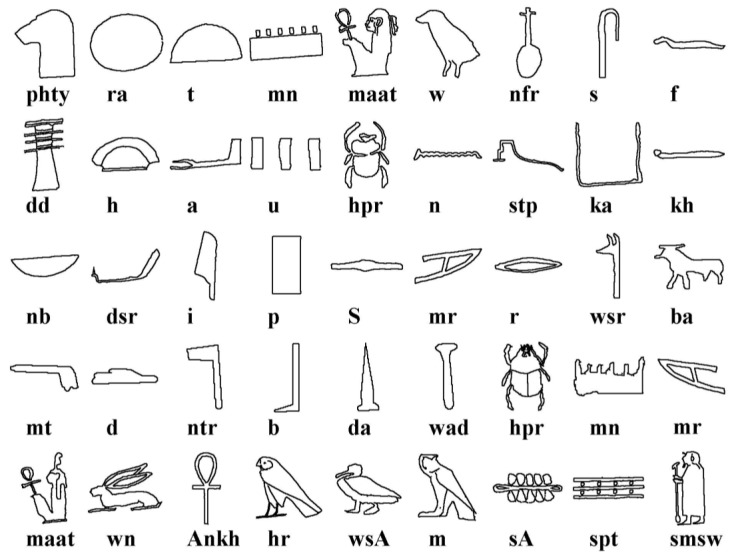
Database of hieroglyphs obtained from the Abydos King List.

Figure 12 shows a set of hieroglyphs and their equivalent phonetic transliteration, as generated by [1]. Bearing this table in mind and considering the symbols obtained by our algorithm, from Figure 11 the following sequence is obtained: RA (Circle), MN (Senet board), PHTY (Leopard head), T (Bread bun), T (Bread bun). The name of the king is RA-MN-PHTY-T-T (Ramesses I).

## 3. Results

This section presents the results obtained by the method presented in the previous section. Aiming to test our system, 261 images of cartouches were used: 76 images obtained from the Abydos King List, 109 images selected from the dataset in [5], and 76 images from other Egyptian monuments and archaeological excavations. The Abydos King List, also known as the Abydos Table, is a list of the names of seventy-six kings of Ancient Egypt.

Although it was found on a wall in the Temple of Seti I at Abydos (Egypt), the table is currently exhibited at the British Museum in London (UK). The dataset of [5] was built from ten pictures of the hieroglyphs found in texts covering the walls of the burial chambers of the Pyramid of Unas at Saqqara (Egypt).The proposed approach has been tested using 1785 hieroglyphs corresponding to the processed cartouches. For the validation of the hieroglyph recognition process, 743 hieroglyphs from the Gardiner’s sign list [1] were used. Table 2 shows information related to some of the evaluated cartouches, containing: inventory number, cartouche drawing, phonetic transliteration, and royal name and dynasty of the king. Intermediate and final visual results, corresponding to these cartouches, are shown in Table 3.

**Figure 13:**
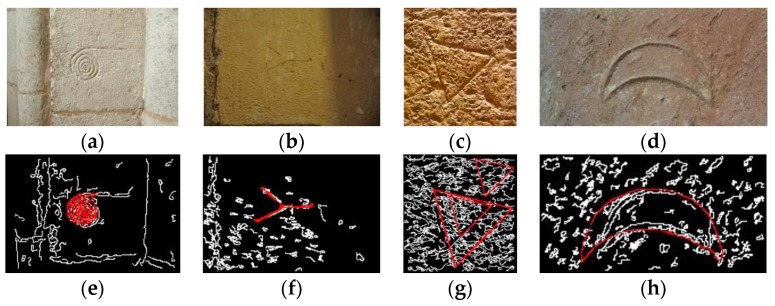
The location process applied to search for stonemason’s marks: (**a**–**d**) original images; (**e**–**h**) localization process results.

Table 4 displays the averaged percentage of errors and standard deviations for the processed images. Because of the differences during the acquisition process and for comparative purposes, results obtained with images from the Abydos King List and the Egyptian Hieroglyph Dataset in [5] are presented separately. Also for comparative purposes, results obtained after each of the three stages of the process are displayed. Images from the Abydos King List and Egyptian Hieroglyph Dataset [5] presented the cartouche conveniently orientated. This makes easier the localization process as one can see from the obtained results. The rest of images used in this experiment do not present the cartouche individually. For this reason, the localization process of the cartouche in the image becomes more difficult than the previous one. Nevertheless, the average rate of success is 89.5. For the whole set of images used in this work, the average rate of success of the localization process is 95.4. Furthermore, the proposed localization process is also valid to recognize some other elements, such as stonemason’s marks as can be seen in Figure 13.

The average rate of success obtained with the second stage (extraction and identification process) is 87.1. It outperforms the hieroglyphs recognition approach described in [5].

**Figure 14:**
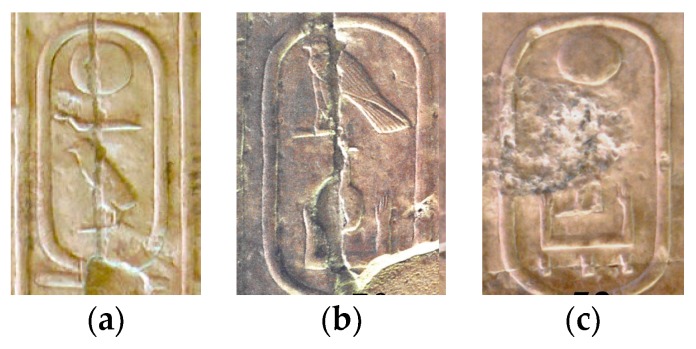
The effects of time, exposure and even vandalism make the recognition process difficult: (**a**) Khufu’s cartouche; (**b**) Neferkahor’s cartouche; (**c**) Qakare Ibi’s cartouche.

Bad preservation of hieroglyphs is the most probably cause of error increase. In fact, the majority of the hieroglyphics that have survived until the present day have suffered the effects of time, weather exposure and even vandalism (Figure 14 shows a set of cartouches).

The last stage of the method and the most important one is the recognition process. In this case, the average rate of success reached with the proposed method is 92.3. Although some hieroglyphs were not properly identified at second stage, it did not affect the recognition process because it took into account the whole hieroglyphs of the cartouche (see the results of Sekhemkhet and Amenhotep III cartouches in Table 3). Thus, properly identified hieroglyphs favor the recognition of the cartouche to the detriment of not acceptably identified hieroglyphs.

The proposed method is able to increase the quantity of hieroglyphs without having the necessity of a training process. Low quality images produce errors in the recognition because of noise. In that case, the same hieroglyph with different changes may be introduced to reduce the error. Common methods to recognize Latin texts were revised but do not work well when there is noise in the image. Therefore, it is difficult to extract the regions, calculate descriptors or moments and use decision-making methods as proposed in previous works [12,13].

One advantage of the proposed method is that the color of hieroglyphs and background can be homogeneous. Besides this proposal avoids the problem of hieroglyphs that cannot be detected with threshold. Previous works revised at Section 1 have considered threshold as the base to extract regions of interest.

An initial drawback of this method was processing time. Nevertheless, to solve this issue some modifications could be considered. To calculate the orientation, a table containing all of them was created. This increases the speed of processing. Several threads can be used to look for each hieroglyph.

## 4. Conclusions

This work presents an automatic method for hieroglyph deciphering that has been conceived to segment and identify the Egyptian characters contained in a cartouche. The proposed three-stage method takes into account several parameters, like the distance between points, and the orientation and the continuity of the edges. Although other techniques have been examined and applied to similar problems, this method has proven to be insensitive to the intensity and the completeness of the objects as well as variable lighting conditions. Promising results have been obtained from this study, delivering good results in terms of image analysis and Egyptian characters’ identification.

**Figure 15:**
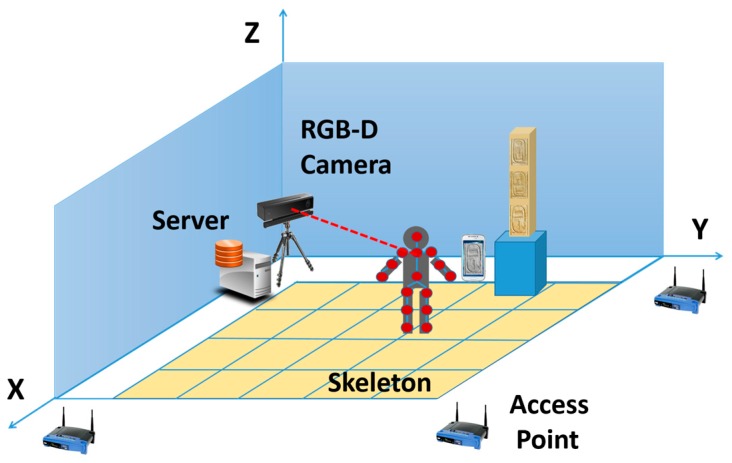
Components of the system.

Aiming to deliver a new strategy for enriching the experience of users visiting a museum, this method can be combined with a positioning system. According to the approach presented in [31], a combination of sensors of different nature, such as RGD-B cameras and WiFi positioning systems (as shown in Figure 15), are employed to accurately locate users in an interior environment. Every access points (i.e., routers) spread in the facilities sends wireless signals that are received by the portable devices—usually smartphones—carried out by users. The Received Signal Strength Indication (RSSI) in each point is used to estimate the position of people in the museum. The identification of 3D skeletons by means of RGB-D cameras is combined with WPS data, making more precise this positioning.

RGB-D sensor used is based on a time-of-flight technology (ToF), Kinect v2. This device delivers up to 2 MPx images (1920 × 1080) at 30 Hz and 0.2 MPx depth maps with a resolution of 512 × 424 pixels. This Kinect camera is connected to a web server where data is saved and processed. The horizontal field of view of the RGB-D sensor is 70 degrees so it is only able to detect people in a section of the room. This section has a size of 3.71 × 3.71 m.

On the other hand, the cellphones employed for localization are also used to take pictures of the objects where the hieroglyphs are written. The processing of these pictures is detailed in this paper.

Although some trials have been already carried out in a controlled environment, the test of the whole infrastructure under more complex and variable circumstances will be performed in the near future, mounting the positioning system in a real gallery with several people visiting the room.

## Figures and Tables

**Table 1:** Results of comparing Chamfer and Hausdorff distances.

Hierogyphs Compared	Chamfer Distance	Hausdorff Distance
*A-B*	36888 (1)	75 (0.52)
*A-C*	21485 (0.58)	144 (1)
*A-D*	16069 (0.43)	35 (0.24)

**Table 2:** List of selected cartouches for visualization purposes.

Abydos Number	Cartouche	Phonetic Transliteration	Royal Name (Dynasty)
17	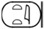	T-t-i	Sekhemkhet (III Dynasty)
22	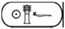	Djed-f-ra	Djedefre (IV Dynasty)
70	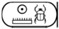	Mn-kheper-ra	Thutmose III (XVIII Dynasty)
72	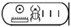	Mn-kheper-u-ra	Thutmose IV (XVIII Dynasty)
73	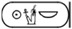	Nb-maat-ra	Amenhotep III (XVIII Dynasty)
75	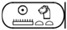	Mn-peht-y-ra	Ramesses I (XIX Dynasty)

**Table 3:** Intermediate and final visual results of six images.

	Sekhemkhet	Djedefre	Thutmose III	Thutmose IV	Amenhotep III	Ramesses I
Original picture	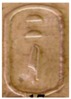	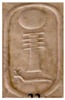	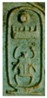	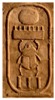	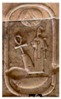	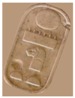
Edge detection	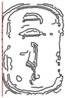	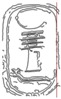	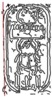	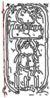	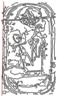	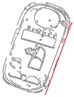
Cartouche localization	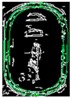	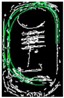	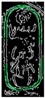	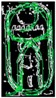	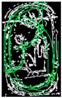	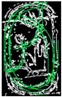
Hieroglyphs extraction	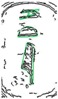	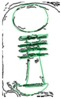	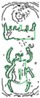	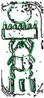	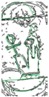	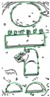
Hieroglyphs identification	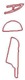	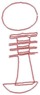	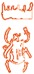	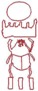	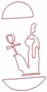	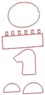
Corresponding cartouche	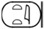	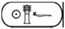	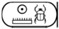	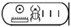	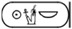	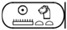

**Table 4:** Average rate of success and standard deviations obtained through the proposed three-stage method.

	%	σ
**Stage 1. Localization process**	**95.4**	**0.4**
Abydos King List dataset	100	-
Egyptian Hieroglyph Dataset [5]	96.3	0.3
Rest of images	89.5	0.9
**Stage 2. Extraction and identification process**	**87.1**	**1.4**
Abydos King List dataset	92.8	1.1
Egyptian Hieroglyph Dataset [5]	84.2	1.6
Rest of images	83.4	1.5
**Stage 3. Recognition process**	**92.3**	**0.9**
Abydos King List dataset	100	-
Egyptian Hieroglyph Dataset [5]	92.7	1.2
Rest of images	84.1	1.5
